# Effective Crizotinib schedule for an elderly patient with ALK rearranged non-small-cell lung cancer: a case report

**DOI:** 10.1186/s13104-015-1126-8

**Published:** 2015-04-23

**Authors:** Aya Fukuizumi, Akihiko Miyanaga, Masahiro Seike, Yasuhiro Kato, Shinji Nakamichi, Kumi Chubachi, Masaru Matsumoto, Rintaro Noro, Yuji Minegishi, Shinobu Kunugi, Kaoru Kubota, Akihiko Gemma

**Affiliations:** Department of Pulmonary Medicine and Oncology, Graduate School of Medicine, Nippon Medical School, 1-1-5 Sendagi, Bunkyo-ku, Tokyo, 113-8602 Japan; Department of Analytic Human Pathology, Graduate School of Medicine, Nippon Medical School, Tokyo, Japan

**Keywords:** EML4–ALK, Crizotinib, Non-small-cell lung cancer, Congestive heart failure, Schedule

## Abstract

**Background:**

Non-small-cell lung cancers (NSCLCs) harboring translocations in anaplastic lymphoma kinase (*ALK*) are highly sensitive to small-molecule ALK kinase inhibitors, such as crizotinib.

**Case presentation:**

We describe a case of post-operative local recurrence of lung adenocarcinoma in an 81 year-old male. He underwent radiation and received chemotherapy with docetaxel, but neither treatment regimen was effective. Following identification of ALK rearrangements, crizotinib treatment was initiated. After treatment with crizotinib for 5 days, adverse events including acute renal failure (grade 2/CTCAE ver4.0) and congestive heart failure (grade 3) occurred. Crizotinib modified treatment was required. Half dose of crizotinib treatment could not control tumor progression. Ultimately, crizotinib was administrated at a dose of 250 mg twice daily every 3 day dosing for 13 months with maintenance of the anti-tumor effect.

**Conclusion:**

This is the first case report that skip schedule was more effective than dose reduction daily in crizotinib administration for ALK rearranged NSCLC patient with severe adverse events.

## Background

EML4-ALK (echinoderm microtubule-associated protein-like 4 and the anaplastic lymphoma kinase) was recently identified as a transforming fusion gene in non-small-cell lung cancer (NSCLC) [1,2]. Approximately 2-7% of patients with NSCLC have rearrangements in the ALK gene, leading to an oncogene-addicted state from aberrant ALK activation [1,3]. Preclinical and clinical studies have shown that cancer cells harboring EML4-ALK are highly sensitive to ALK inhibition [4,5].

Crizotinib is the first clinically available ALK tyrosine kinase inhibitor (TKI) and competes with ATP for binding to the tyrosine kinase pocket of the enzyme, inhibiting tyrosine phosphorylation of activated ALK at nanomolar concentrations. The most common adverse events associated with crizotinib include visual disturbances, gastrointestinal symptoms, peripheral edema, fatigue, decreased appetite, and elevated aminotransferases [4,6]. The majority of the toxicities are grade 1 or 2, and some improve with continuation of therapy. Here, we present for the first time a case in which every 3 day dosing administration of crizotinib was effective and prevented adverse events in elderly NSCLC harboring the ALK fusion gene.

## Case presentation

**Figure 1:**
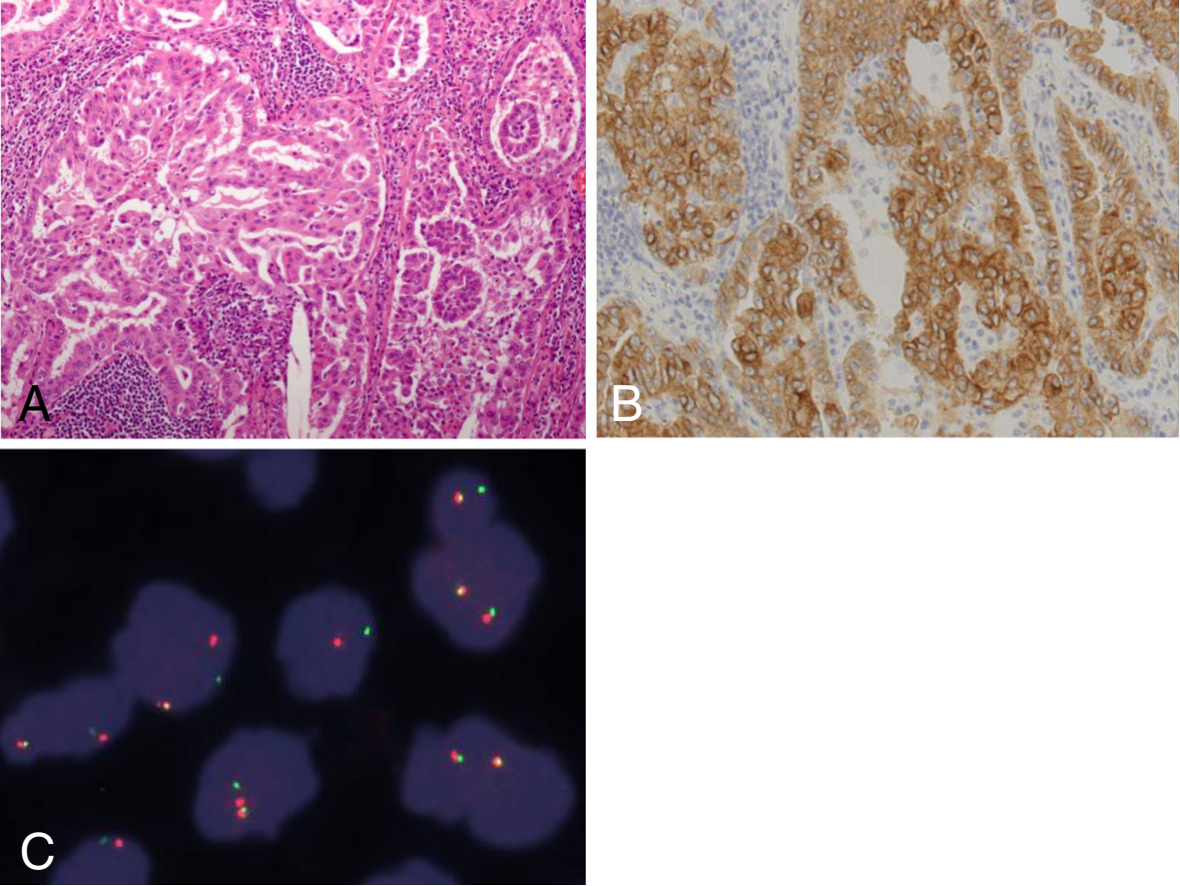
Histological and immunohistochemical findings in the primary tumor: **(A)** adenocarcinoma with mixed papillary and acinar subtype (hematoxylin and eosin 40× magnification), **(B)** Immunohistochemical analysis of the ALK protein expression in the tumor cells, **(C)** Fluorescence in situ hybridization (FISH) analysis of *EML4-ALK.*

A 81 year-old male former-smoker who experienced previously acute myocardial infarction presented in January 2013 with mediastinal lymph node metastasis. He was diagnosed as lung adenocarcinoma (AC) seven years ago and underwent right-middle lobectomy and lymph node-dissection (ND2a-1). His pathological stage was stage IA (pT1bN0M0). The histopathological subtype of the specimen was mixed papillary and acinar subtype AC components (Figure 1A). Immunohistochemistry showed positive TTF-1, PE10, CD7, and Alcian blue & PAS staining. In 2011, however, a fluorine 18-labled fluorodeoxyglucose (FDG)-positron emission tomography-computed tomography (PET-CT) scan showed mediastinal lymph node metastasis. Although local recurrent mediastinal radiation therapy (52 Gy) was performed, the mediastinal lymph metastasis progressed. Therefore, in January 2013 the patient received one cycle of chemotherapy with docetaxel in consideration of elderly and the co-morbities. However, the treatment was withdrawn because of serious upper gastrointestinal bleeding due to the development of hemorrhagic gastric ulcer during his medical course.

**Figure 2:**
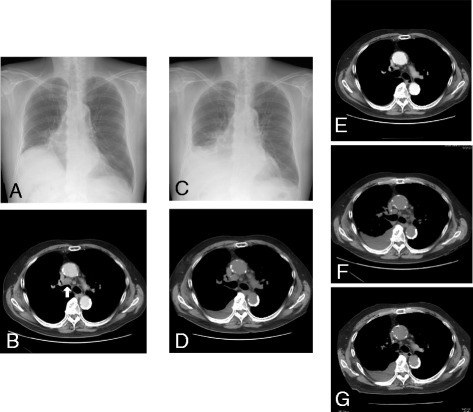
Chest radiograph and chest CT scan before **(A** and **B)** and after **(C**, **D**, **E**, **F** and **G)** crizotinib treatment: **(A)** Chest radiograph before crizotinib therapy, **(B)** and swelling of the mediastinal lymph node (#4R), **(C)** chest radiograph after 5 days of crizotinib treatment revealing increase in pleural effusions, **(D)** detection of right pleural effusions due to the appearance of congestive heart failure and renal failure, **(E)** disappearance of pleural effusions and significant shrinking of the mediastinal lymph node. **(F)** CT scan just before the crizotinib re-challenge after 4 months withdrawal. **(G)** CT scan after 3 months of the crizotinib re-challenging treatment.

**Figure 3:**
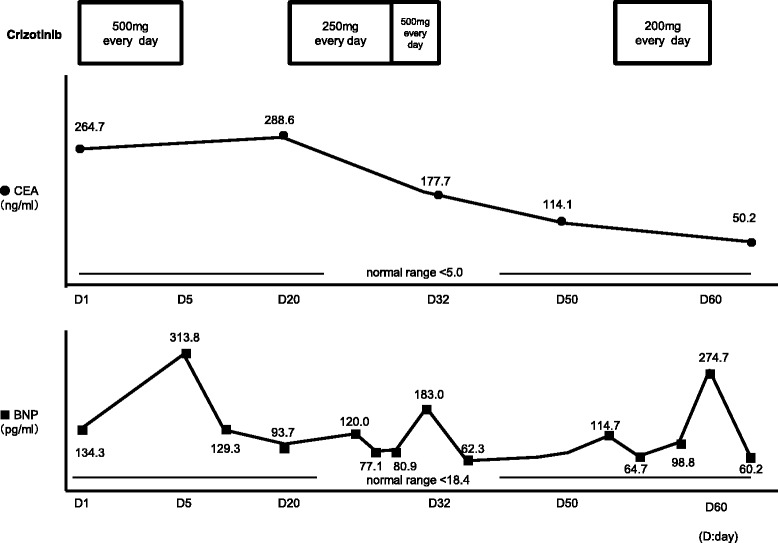
Changes in serum CEA and BNP levels after the resumption of firstly crizotinib therapy.

Mutation analysis revealed that the tumor expressed the wild type epidermal growth factor receptor (*EGFR*) gene. However, *ALK* rearrangements were identified using fluorescent in situ hybridization (FISH) and confirmed by immunohistochemistry for ALK expression in both tumors (Figure 1B and C). Performance status (PS) was good (PS 1). We assessed that cytotoxic chemotherapy was high risk because of short time from his recovery from the upper gastrointestinal bleeding. Therefore, crizotinib was administered orally at a dose of 250 mg twice daily as a second-line treatment (Figure 2A and B). After 5 days of crizotinib therapy, the patient developed acutely deteriorating dyspnea and exhibited arterial oxygen desaturation and hypoxemia (SpO2 89% in room air). Chest radiograph and CT scan revealed increase of right pleural effusions (Figure 2C and D). Serum B-type natriuretic peptide (BNP) was increased from 134 to 314 pg/ml (Figure 3), and creatinine (Cr) was elevated from 1.18 to 1.55 mg/dl. The electrocardiogram was normal, showing no evidence of arrhythmia, or acute myocardial infarction. Increased levels of high sensitive Trop-T (0.024 ng/ml) and CK-Mb (<4.0 ng/ml) were not found. Cardiac ultrasonography revealed no abnormality (Ejection Fraction 78%). According to the Framingham Study, we diagnosed his clinical manifestation, congestive heart failure and acute renal failure. Since side effects of crizotinib were suspected, the drug was immediately discontinued and started with furosemide 10 mg/day. Oxygenation, serum creatinine and BNP immediately improved (Figure 3). Therefore, crizotinib administration was resumed at 250 mg/day combined with furosemide 10 mg/day.

**Figure 4:**
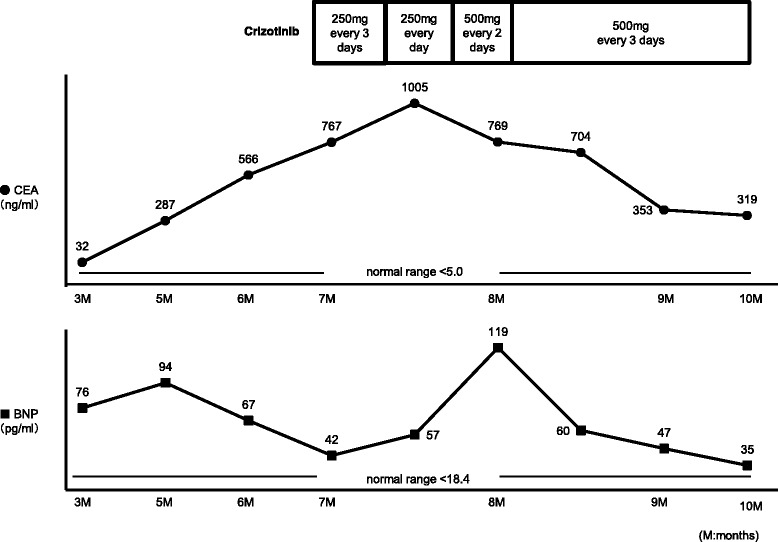
Changes in serum CEA and BNP levels after the rechallenge of crizotinib therapy.

Since there were no adverse events for 10 days, the crizotinib dose was increased to 250 mg twice daily. By following this regimen, the patient achieved significant tumor response, as shown by CT scan (Figure 2E). However, he complained of chest discomfort again after 3 days and crizotinib therapy was once again discontinued. One month later, the patient was re-challenged at a low dose of 200 mg/day crizotinib therapy. Despite the dose reduction, pleural effusion and exacerbation of creatinine (1.96 mg/dl) and BNP (274.7 pg/ml) occurred on day 11. Four months later, as he had increased pleural effusion and lymph node metastasis (Figure 2F), he was re-challenged with 250 mg/day crizotinib administered every 3 days; however, Carcinoembryonic antigen (CEA) was elevated (Figure 4). Once again 250 mg/day drug was prescribed, but no anti-tumor effect was observed. Administration of 250 mg twice daily was then tried on every other day, but BNP levels increased (Figure 4). Finally, the frequency was reduced to 250 mg twice daily every 3 day dosing, and both BNP and CEA levels were decresed (Figure 4). Finally, a significant response was achieved without adverse effects (Figure 2G). This treatment has been continued with good response for 13 months.

## Discussion

We report a rare case in which acute renal failure and congestive heart failure occurred after the administration of crizotinib for lung AC with *ALK* rearrangement, and in which dose and schedule modification were required. Because elevations in serum BNP, Cr and pleural effusions were reversible on discontinuation of crizotinib, these were recognized as adverse effects of crizotinib. To our knowledge, no adverse effects on congestive heart failure patients have been described with crizotinib therapy in previous clinical trials [4,6]. Renal toxicity of crizotinib has previously been described (<10%) [7]. However, the patient concerned was elderly and had exhibited a decline in cardiac function following an occurrence of acute myocardial infarction 10 years ago. By repetition of capacity modulation, we have demonstrated the effectiveness of continuing crizotinib therapy at a dose of 250 mg twice daily every 3 day dosing.

In EGFR-TKI treatment for advanced NSCLC with EGFR mutation, some patients need a dose reduction of gefitinib due to occurrence of several adverse effects. A treatment strategy minimizing toxicity is especially required for patients in poor condition, or for elderly patients. A previous report demonstrated that low-dose gefitinib may not be inferior to standard-dose gefitinib for NSCLC patients with *EGFR* mutations [8]. On the other hand, there are no reports of NSCLC patients with *ALK* in whom successful clinical benefit without adverse effects was observed by administration of crizotinib on alternate days. Regarding a treatment schedule for crizotinib, a previous case reported progression of brain metastases under the use of standard crizotinib therapy (250 mg twice daily); however, readjustment of crizotinib dose to 500 mg once daily generated a favorable response [9]. These authors hypothesized that increasing the maximum plasma concentration (Cmax) without increasing the daily dose of 500 mg could be beneficial.

Regarding pharmacokinetic-pharmacodynamic (PKPD) simulation of crizotinib treatment, while Cmax was 243 ng/ml for a once daily dose of crizotinib 400 mg, it was 493 ng/ml for a standard schedule of 250 mg twice daily [10]. Taking into account the fact that the plasma half-life was 29.1 hours for crizotinib 400 mg once daily, an effective plasma concentration was preserved, since every 3 day dosing administration of 250 mg twice daily may have prevented excessive elevation of Cmax.

## Conclusions

The present report indicates that alternate day administration of crizotinib is a choice worth considering, since it maintains sufficient clinical effects, while avoiding serious adverse events, especially in elderly and high-risk cases. Every 3 day dosing administration may be an option but only in patients intolerant of standard dosing trial.

Therefore, it is necessary to study the relationship between pharmacokinetics of crizotinib with every 3 day dosing administration and clinical effects, as well as standard administration.

### Ethics approval and consent

Clinical assessment and genetic testing in this patient were performed primarily for diagnostic purposes. The patient gave his informed consent to diagnostic genetic testing. Written informed consent was obtained from the patient’s family for publication of this case report and accompanying images due to the death of the patient himself before publication.

## Abbreviations

NSCLC: Non-small-cell lung cancer
EML4: Echinoderm microtubule-associated protein-like 4
ALK: Anaplastic lymphoma kinase
TKI: Tyrosine kinase inhibitor
AC: Adenocarcinoma
PET-CT: Positron emission tomography-computed tomography
EGFR: Epidermal growth factor receptor
FISH: fluorescent in situ hybridization
PS: Performance status
BNP: B-type natriuretic peptide
Cr: Creatinine
CEA: Carcinoembryonic antigen
